# The Utility of Genomic and Transcriptomic Data in the Construction of Proxy Protein Sequence Databases for Unsequenced Tree Nuts

**DOI:** 10.3390/biology9050104

**Published:** 2020-05-19

**Authors:** Cary Pirone-Davies, Melinda A. McFarland, Christine H. Parker, Yoko Adachi, Timothy R. Croley

**Affiliations:** 1 Office of Regulatory Science, Center for Food Safety and Applied Nutrition, U.S. Food and Drug Administration, College Park, MD 20740, USA; Melinda.McFarland@fda.hhs.gov (M.A.M.); Christine.Parker@fda.hhs.gov (C.H.P.); Timothy.Croley@fda.hhs.gov (T.R.C.); 2 Joint Institute for Food Safety and Applied Nutrition (JIFSAN), University of Maryland, College Park, MD 20740, USA; 3 Office of Analytics and Outreach, Center for Food Safety and Applied Nutrition, U.S. Food and Drug Administration, College Park, MD 20740, USA; Yoko.Adachi@fda.hhs.gov

**Keywords:** nut allergen, walnut, pecan, *Juglans regia*, de-novo transcriptome, proteomics, database

## Abstract

As the apparent incidence of tree nut allergies rises, the development of MS methods that accurately identify tree nuts in food is critical. However, analyses are limited by few available tree nut protein sequences. We assess the utility of translated genomic and transcriptomic data for library construction with *Juglans regia*, walnut, as a model. Extracted walnuts were subjected to nano-liquid chromatography–mass spectrometry (n-LC-MS/MS), and spectra were searched against databases made from a six-frame translation of the genome (6FT), a transcriptome, and three proteomes. Searches against proteomic databases yielded a variable number of peptides (1156–1275), and only ten additional unique peptides were identified in the 6FT database. Searches against a transcriptomic database yielded results similar to those of the National Center for Biotechnology Information (NCBI) proteome (1200 and 1275 peptides, respectively). Performance of the transcriptomic database was improved via the adjustment of RNA-Seq read processing methods, which increased the number of identified peptides which align to seed allergen proteins by ~20%. Together, these findings establish a path towards the construction of robust proxy protein databases for tree nut species and other non-model organisms.

## 1. Introduction

The term “tree nuts” refers to a group of phylogenetically diverse, edible seeds that grow on trees and includes walnut, pecan, almond, and hazelnut, among others. Tree nuts are widely consumed and offer high nutritional value and additional health benefits [1]. However, they are also one of the most common foods to cause allergic reactions, with tree nuts alone responsible for 18–40% of anaphylaxis cases [2,3,4]. The prevalence of tree nut allergies varies based on geography, age, and the definition used for diagnosis, but ranges from 0.05–7.3% of the population [4]. Thus, the accurate detection of tree nuts in food is essential for consumer safety.

Enzyme-linked immunosorbent assay (ELISA) is the standard method for the detection of tree nuts in food. Although ELISA is sensitive and convenient, results from different commercial kits can vary [5], and assay specificity may be compromised by cross-reactivity with closely related species [6]. Liquid-chromatography–mass spectrometry (LC-MS/MS) offers a high degree of sensitivity and specificity and can identify multiple targets in a single sample. Thus, MS provides a versatile solution for allergen detection in complex food samples and can also be used to verify ELISA results. 

In proteomic experiments, the selection of a protein sequence library is a key component of data analysis. Typically, tryptic peptides are identified and their associated proteins inferred from searches of MS/MS spectra against a library that contains protein sequences, which are identical or highly similar to sample proteins [7]. A proteome, the set of proteins derived from the bioinformatic annotation of a genome, is often used to provide such a comprehensive protein set. However, a proteome or other complete set of proteins is not always available. This is particularly true when working with non-model species such as those categorized as tree nuts and is demonstrated by the paucity of tree nut protein sequences available in Uniprot and Genbank [8]. 

MS/MS search results can be greatly improved by the use of translated genomic and transcriptomic data to supplement existing protein sequences for database construction [9,10,11]. Such translated databases are often referred to as proteogenomic databases. Sample-specific RNA-Seq databases yield search results which approximate those of proteomic databases [12]. This proteogenomic approach is useful when protein data are sparse but can also increase peptide and protein identifications when a proteome is available [12,13,14]. For example, a transcriptome database may facilitate the identification of sequences containing single amino acid variations (SAVs) [14,15,16] or RNA-splice and editing variants not present in the proteome [17]. Searches against a six-frame translation of a genome may identify novel peptides located within introns, across exon–intron boundaries, or in unannotated regions of the genome [9,10,11]. Less information is available regarding the impact that proteomes from different annotation pipelines have on mass spectrometry searches. With the rise of next-generation sequencing and analysis, multiple proteomes may be available for some species. 

Despite the proven utility of proteogenomic databases, few studies have focused on plants, and none have focused on tree nut species. Thus, we created five custom databases from publicly available data for English walnut, *Juglans regia,* including a six-frame translation (6FT) of the genome, three from proteomes generated by different pipelines analyzing the same genome, and a tissue-specific de-novo-assembled transcriptome. *J. regia* is one of the few tree nut species for which high quality genomic and transcriptomic data are available. We performed nano-LC-MS/MS on the tryptic digests of the nuts of *J. regia* and searched the resultant spectra against the databases. We evaluated the utility of these data for improving MS/MS searches.

In addition, we determined methods to improve de-novo transcriptome quality and downstream peptide identification. RNA-Seq pipelines consist of several read processing steps, namely, read quality trimming, error correction, and normalization, followed by assembly. The majority of proteomics studies rely on the program Trinity to assemble RNA-Seq reads [18] which is consistently ranked as one of the best de-novo transcriptome assemblers [19,20,21,22]. Thus, we utilized Trinity for assembly and focused on adjusting read processing methods, including quality trimming, error correction, and normalization to improve MS/MS searches. To our knowledge, there are no other studies that examine the effects of these methods on proteogenomic database performance.

## 2. Materials and Methods

Tryptic digests of ground and defatted raw walnut seeds were extracted and subjected to n-LC-MS/MS on an Orbitrap Elite mass spectrometer (Thermo Scientific, San Jose, CA) coupled to a nanoACQUITY UPLC system (Waters, Milford, MA) (*n* = 2). Samples were run in triplicate. Raw data files were converted to peak list files using Proteome Discoverer v. 2.2 (Thermo Scientific, San Jose, CA) and searched against custom databases using Mascot v. 2.5 (Matrix Science, London, UK) [23]. Search parameters included tryptic peptides with a precursor ion mass tolerance of 20 ppm, a fragment ion mass tolerance of 0.8 Da, the allowance of two missed cleavages, a fixed modification of carbamidomethyl (cysteine), and a variable modification of the oxidation of methionine. Peptides were accepted as correctly identified if the ion score was greater than the identity score (*p* < 0.05). Search results from all six raw data files were pooled, and peptides were compared across databases using MassSieve v. 1.14 [24]. Peptides with less than two spectral counts and peptides from proteins with less than two supporting peptides were removed at the experiment level.

All databases were constructed from publicly available data and derived from the published *J. regia* genome (Genbank Nucleotide: LIHL01000010.1) [25], except the transcriptome, which was generated independently by the same group [25]. Three different proteomes [26], predicted using three different annotation pipelines on this genome [26,27,28], were downloaded. These included the NCBI Proteome (GCF_001411555.1), the published Maker proteome [25], and the Braker proteome [29] deposited in the TreeGenes database [30].

To prepare a six-frame translation of the genome, the perl script splitter.pl, available through Mascot, was used with default parameters to split the genome into chunks, and a six-frame translation was generated within the Mascot engine. A de-novo transcriptome was assembled from RNA-seq reads from the walnut embryo and immature fruit (~48 million paired-end reads, length = 85 bp) [25]. Reads were downloaded from TreeGenes and FastQC [31] was used to assess data quality. To remove reads containing ribosomal RNA (rRNA) sequences, all reads were aligned to sequences in the SILVA database [32] using bowtie2 2.3.1 [33], and only those which did not align were included in downstream analyses. Adapters were removed with BBDuk [34], part of the BBTools package developed by the Joint Genome Institute (JGI, Berkeley, CA). Sequences were quality trimmed to phred 35 using Trimmomatic [35], and Trinity v2.5.1 with default parameters was used to normalize and assemble reads. Trimming stringency, normalization and assembly followed the methods of Martinez-Garcia et al. (2016), except that a more recent version of Trinity (2.5.1 vs. 2.0.6) was utilized [25] which included a max_pct_stdev parameter setting of 10,000. Transdecoder [36] was used to convert the nucleotide transcript sequences to amino acids and obtain open reading frames (ORFs) from all six reading frames. Duplicate sequences with 100% sequence identity were removed with CD-HIT [37]. Protein sequences for common contaminants were appended to all databases except the genome [38].

**Figure 1:**
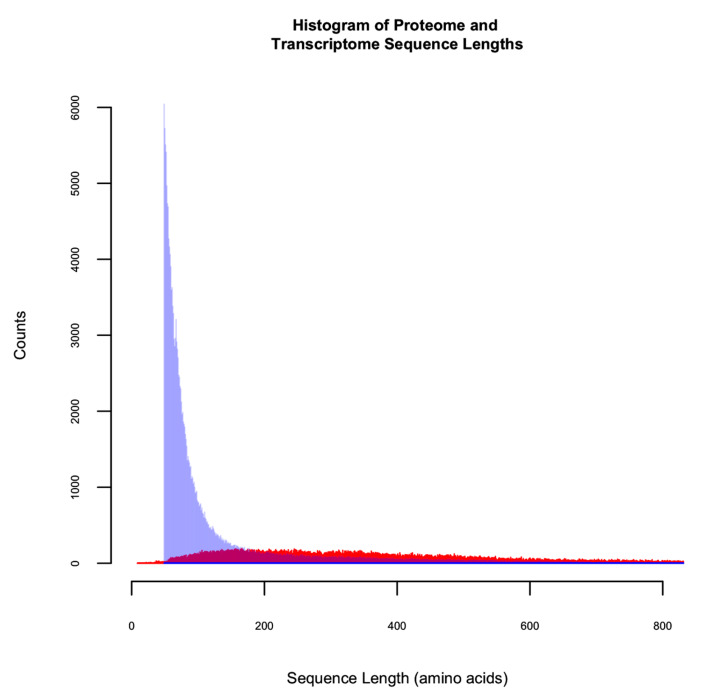
Histogram of the sequence lengths of the NCBI proteome and the transcriptome. The transcriptome is represented in blue, the proteome in red.

To accurately compare the sequence content of each database and minimize the effect of variable database size on probability-based scoring, all databases except the genome were sized to match the largest database, the transcriptome. To accomplish this, bacterial sequences were appended to each walnut database until the total number of combined residues (walnut plus bacterial) matched that of the translated transcriptome, 26,627,682. Sizing was based on the number of transcriptome residues, not the number of sequences, due to the discrepancy in the distribution of the sequence lengths from the transcriptome and proteomic databases (Figure 1). The translated transcriptome is comprised of sequences that are greater in number and, on average, shorter than the proteome sequences. For a discussion of the causes of this discrepancy, please see the third paragraph of the discussion.

To improve RNA-Seq read processing for peptide identifications from MS/MS searches against a transcriptomic database, the same set of tissue-specific walnut RNA-Seq reads as above were utilized. Trimmomatic was used to trim reads to either phred 35 or phred 5, and Rcorrector [39] was used to correct erroneous kmers in some assemblies. In addition, the python script FilterUncorrectabledPEfastq.py, available from the Harvard Informatics GitHub repository TranscriptomeAssemblyTools, was used to remove sequences containing errors deemed “unfixable” by Rcorrector. Sequences were normalized and assembled using the Trinity package [18]. As above, ORFs in all six reading frames were extracted using Transdecoder, and sequences with 100% sequence similarity were removed using CD-HIT. This resulted in the creation of 32 transcriptomic databases (eight read processing conditions, each with four replicates). Data were normalized and assembled for each replicate using Trinity. A complete workflow for database construction is outlined in Figure S1. Performance of transcriptomic databases was compared as above, using Mascot and MassSieve. The numbers of peptides identified when searched against each walnut transcriptomic database were compared using a one-way analysis of variance (ANOVA) using Tukey’s adjustment for multiple comparisons. Both analyses were implemented in R [40], and statistical significance was evaluated at alpha equal to 0.05. As a second dataset, ~310 million reads (paired-end, 100 bp) from four stages of nut development of *Carya illinoinensis*, pecan [41] were downloaded from the Sequence Read Archive (SRA) [42] at the National Center for Biotechnology Information (NCBI) and processed as the walnut reads. However, reads were quality trimmed to phred 30 instead of phred 35. A large proportion of the reads scored between phred 30–35, thus phred 30 was selected to retain any unique information contained within those reads.

The quality of de-novo transcriptome assemblies was assessed using the N50 value, the total number of transcripts, and the total number of assembled bases, which were calculated using the perl script Trinity_stats.pl bundled with the Trinity package. The percentage of reads that aligned to the assembly was assessed using bowtie2 [33]. Contiguity was estimated by calculating the number of walnut reference proteins from the NCBI proteome that were covered along at least 90% of their length by at least one transcript (sequence similarity = 100%). Calculations were executed using the trinity script analyze_blastPlus_topHIt_coverage.pl bundled with the Trinity package.

## 3. Results

### 3.1. The Utility of Databases Built from a 6FT of the Genome, Three Proteomes, and a Six-Frame Translation of a Transcriptome Assembled Using Default Parameters

To assess the utility of genomic and transcriptomic data for the construction of MS protein databases in walnut, we searched spectra resulting from the MS analysis of raw *J. regia* walnut samples against five custom databases built from three proteomes, a six-frame translation of the genome, and the translated transcriptome (reads were trimmed to phred 35 and assembled using Trinity with default parameters [25], but with Trinity v2.5.1). In order to minimize bias in scoring due to database size, all databases were sized to match the largest database, the transcriptome (see methods).

**Figure 2:**
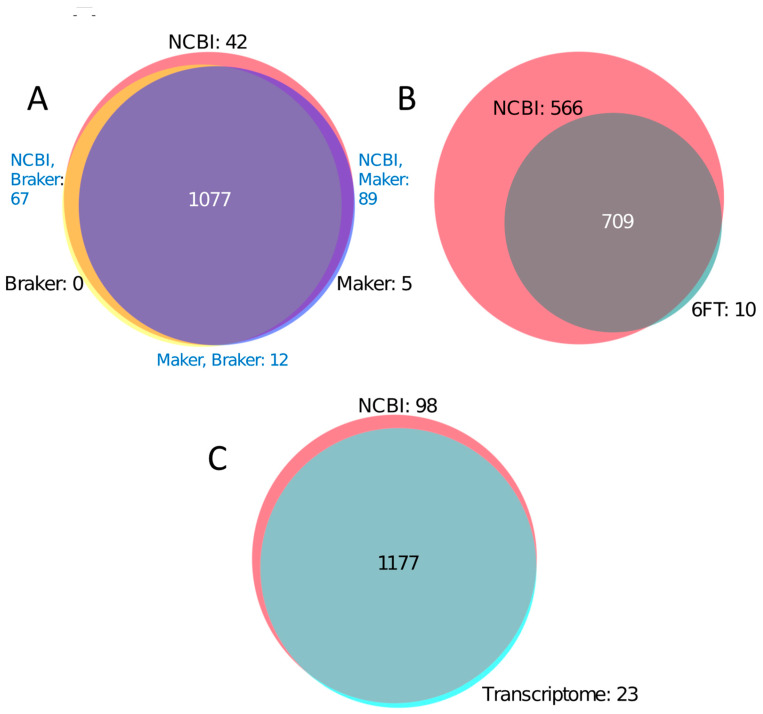
Venn diagrams comparing the total number of peptides identified in five databases. Number of unique peptides listed, along with the number of peptides shared by all databases. (**A**). NCBI, Maker, and Braker databases, (**B**). NCBI and 6FT databases, (**C**). NCBI and Transcriptomic databases.

Three proteomes are publicly available for *J. regia*, each derived from the annotation of the walnut genome [25] by different pipelines (NCBI Eukaryotic Genome Annotation, Maker, Braker). We compared the results of searches against databases constructed from these three proteomes. Searches against the NCBI database identified the greatest number of peptides, followed by those against the Maker and Braker databases (1275, 1183, and 1156, respectively) (Table 1, Figure 2A).

Searches against the NCBI database identified 42 unique peptides not present in the Maker and Braker databases (Figure 2A). Collectively, searches against the Maker and Braker databases identified 17 peptides not present in the NCBI database, 12 of which were identified in both databases, and five of which were identified only in the Maker database. The majority of the sequences of uniquely identified peptides were absent in databases in which they were not identified. However, several such peptide sequences were present in a database but not identified, which was primarily due to the stringent filtering criteria used in this work. The presence or absence of all uniquely identified fully tryptic peptide sequences in all three proteomic databases can be viewed in Table S5. Unique peptides across all databases aligned most frequently to heat shock proteins (HSPs), low-temperature-induced proteins, and seed storage proteins including vicilin-like proteins and 11 s globulins. A list of all identified peptides as well as the most parsimonious set of proteins to which they align can be viewed in Supplemental Table 1. Seed storage proteins with high peptide coverage are critical for the MS/MS identification of tree nut species, as these are the most abundant proteins present in the nut and are targeted for marker development [8]. Thus, we were particularly interested in identifying which database(s) maximize the peptide coverage of these seed storage proteins. Two such peptides were uniquely identified in the NCBI database: TEAGEMR, which aligns to a vicilin-like protein, and GLHGAAIPGCAETFQSESSSQFR, which is present in a legumin-B like protein. Due to the superior performance of the NCBI database, we selected it as the basis for additional comparisons throughout this paper.

To determine whether a database constructed from a 6FT of the genome contributes novel information for the MS/MS analyses of nuts, we next compared results from searches against a 6FT database and the NCBI proteomic database. As expected, a greater number of peptides was identified in searches against the NCBI database than against the 6FT database (1275 vs. 719, respectively) (Figure 2B). However, only ten peptides were uniquely identified in the 6FT database, suggesting that novel peptide types are not relevant for proteomics studies of the nut.

We next determined whether the translated transcriptome can serve as a proxy protein database when a proteome is not available. We compared results from searches against a transcriptomic database constructed from immature fruit and embryo tissue against those from searches that utilized the NCBI proteomic database. Although the NCBI database outperformed the transcriptomic one (1275 vs. 1200 peptides, respectively), the transcriptomic database yielded 92% of the peptides identified in the NCBI database, plus 23 unique peptides (Table 1). Ninety-eight peptides were uniquely identified in the NCBI database.

A transcriptomic database may allow the identification of peptides not present in the proteome, such as those containing single amino acid variations (SAVs) or those present in RNA-splice variants [14,15,16]. The majority of peptides identified only in the transcriptome contain or are flanked by SAVs, and several are present in sequences which are likely protein species. Two unique SAV-containing peptides were identified in seed storage proteins, LYDTSNQANQLDENAR, present in a legumin-B like protein, and TMLGPELAAAFGVSEEK, which aligns to a highly abundant vicilin-like seed storage protein. This number was low compared to the 40 peptides uniquely identified in the NCBI database that are located within seed storage proteins (Table S2).

### 3.2. The Utility of Translated Transcriptomic Databases Assembled Using Varying Read Processing Parameters for Peptide Identifications

**Figure 3:**
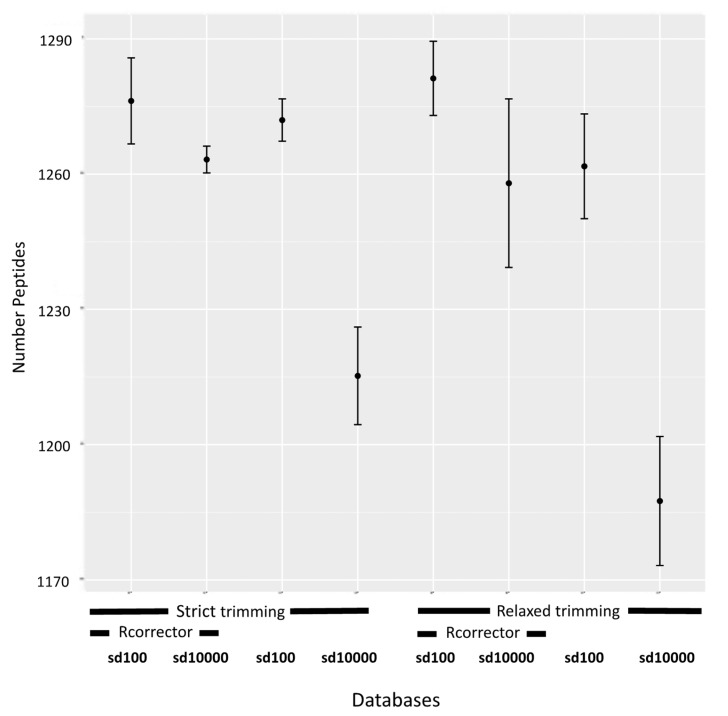
Plot of the mean number of peptides identified across eight transcriptomic databases constructed using different read processing conditions in walnut (*n* = 4). Bars represent standard deviations, sd = max_pct_stdev.

Given the utility of the transcriptomic database as a proxy protein database, we sought to improve its quality in order to more closely approximate or surpass the performance of the NCBI database. Pre-assembly methods minimize sequencing errors and reduce the size and redundancy of the dataset, which can in turn affect assembly quality. The removal of errors is accomplished by discarding low-quality bases or replacing erroneous bases with correct ones, which improves read accuracy and the reliability of downstream data analyses [43]. However, the optimal threshold of quality trimming is debated [44], and error correction is not always utilized [45,46,47]. Normalization, which reduces the number of redundant reads in a dataset, may also impact the error content of reads [48,49]. Our preliminary studies indicated that the parameter max_pct_stdev, which controls the retention of reads with aberrant kmer abundance profiles during Trinity normalization, affects transcriptome quality and the number of peptides identified downstream. Here, we assessed the effects of two values of max_pct_stdev (100 or 10,000), strict and relaxed read quality trimming, and the application of read error correction or not, on the number of identified peptides during MS/MS searches. The total mean number of peptides identified from MS/MS searches against the transcriptomic databases (*n* = 4) are shown in Figure 3. Six of the treatment combinations, those that employed max_pct_stdev equal to 100 and/or Rcorrector, identified a significantly greater number of peptides than the two constructed using max_pct_stdev equal to 10,000 and without Rcorrector (Table S3). Standard quality metrics for transcriptome nucleotide assemblies (Table S4) are in concordance with peptide counts. The transcriptomes used to construct the six high-performing databases are of slightly higher quality than those used to construct the two low-performing ones (Figure S2).

To validate the use of max_pct_stdev equal to 100 and/or the application of error correction in a second dataset, we constructed 12 additional databases from an independently produced set of RNA-Seq reads from the nuts of *Carya illinoinensis*, pecan [41]. Database construction and MS/MS analyses were performed as in walnut, except that only three replicate databases were constructed, and a single trimming threshold was selected. We opted to use strict trimming, as peptide counts were on average slightly higher and transcripts were slightly more contiguous under this condition than when phred 5 was used. As in walnut, databases constructed using max_pct_stdev equal to 100 and/or Rcorrector outperformed the databases constructed without error correction and with max_pct_stdev equal to 10,000 (Figure S3).

**Figure 4:**
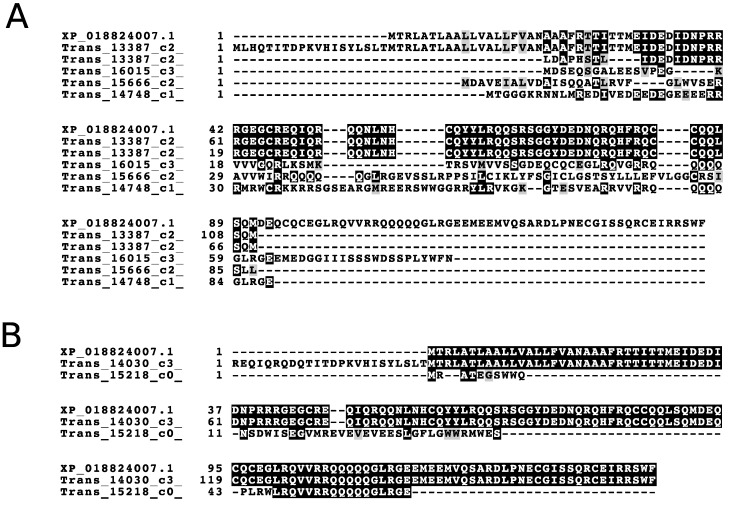
Alignment of sequences from a cluster of related sulfur-rich seed storage sequences from a parsimonious comparison (**A**). Comparison of the NCBI proteome (XP 018824007.1) and the transcriptome assembled under published conditions. (**B**). Comparison of the NCBI proteome and the improved transcriptome assembled using Rcorrector and max_pct_stdev = 100.

We also compared search results from walnut databases constructed from the NCBI proteome, the initial transcriptome used above built using published methods, and a current version of Trinity (strict quality trimming, no error correction, and assembly with Trinity under default parameters, max_pct_stdev = 10,000) [25,47,50], and the improved transcriptome processed using strict quality trimming, error correction, and max_pct_stdev equal to 100 (Data S1). Results from searches against the improved transcriptome and the NCBI database were similar (1281 versus 1275 peptides identified, respectively), and a greater number of peptides were identified in searches against the improved transcriptome than searches against the initial one (1281 versus 1200 peptides, respectively). Of the 81 peptides gained using the improved transcriptome, 37 align to seed storage proteins, an approximately 20% increase in the total number of peptides identified in proteins of this type. In addition, some transcripts are less fragmented in the improved transcriptome than in the initial transcriptome. Figure 4 compares alignments of proteomic and transcriptomic sequences from a sulfur-rich seed storage cluster, including translated transcripts from both the initial (Figure 4A) and improved transcriptomes (Figure 4B). The number of transcript sequences shifted from five highly fragmented sequences in the initial transcriptome to two more complete sequences in the improved transcriptome. One of the improved transcript sequences shares 100% sequence similarity along 100% of the length of XP_018824007.

Lastly, we also performed a search against the NCBI database with bacterial sequences removed in order to assess its performance against the improved transcriptomic database. While searches against the sized NCBI database identified 1275 peptides, searches against the unsized version yielded only a slight increase in the number of identified peptides, 1283, indicating that searches against the improved transcriptome (1281 peptides) and the unsized NCBI database (1283 peptides) also yield similar results.

## 4. Discussion

The identification of variable numbers of peptides when searching against the three proteomic databases demonstrates that genome annotation affects downstream peptide identifications and should be considered during proteomic studies. Genome annotation is complex, and includes ab-initio gene predictions based on mathematical models, as well as evidence-based predictions, which are based on the alignment of independent sequence data to the genome (i.e., RNA-Seq, expressed sequence tag (EST), and proteins from related organisms) [51]. In addition, intron–exon borders must be predicted, a process which can be error-prone [51]. Different annotation pipelines leverage different gene finding algorithms and aligners to complete these tasks. Thus, final gene models may vary between pipelines. For walnut, the annotations produced by the NCBI pipeline yielded the greatest number of peptides, but more work is needed to ascertain the effects of different annotation pipelines in additional studies.

As expected, the 6FT identified fewer peptides than the proteomic database. The majority of peptides from expressed sequences are located within exons or span exon–exon boundaries [9]. Both of these peptide types are present in proteins, but only those located within exons occur in a complete genome. It is also worth noting that search sensitivity decreases as search space increases [52], thus some peptides may not be included in the analysis of peptide identifications from the large 6FT database due to a prohibitively low score. On the other hand, novel peptides from unannotated genes or proteins translated from alternative coding frames may also be present in a genome, as well as those which occupy intergenic regions, introns, untranslated 3′ or 5′ regions, or exon–intron boundaries [9]. Interestingly, only ten unique peptides were identified in the 6FT database, suggesting that these peptide types are not important for MS studies of the nut. However, such a database may be useful in other proteomics studies which seek to detect rare or novel proteins.

When a genome and proteome are not available, our data show that a transcriptomic database can be used as a proxy protein database. The major drawback of utilizing a transcriptome for database construction is the high degree of transcript fragmentation and truncation resulting from RNA degradation during extraction and sequencing, errors introduced during sequencing, and erroneous or incomplete assembly. Incomplete transcripts may decrease the number of identified peptides in two ways. A peptide may not be identified if a portion of the transcript which would otherwise contain the peptide is missing. Alternatively, a peptide may not be included in the final analysis even if present in the transcript, if the transcript is truncated in a way that precludes the identification of at least one additional peptide. In this case, the protein identification is no longer supported by at least two peptides and that protein may be removed from the final results to reduce false positives [53]. From this perspective, a proteomic database which contains predominantly full-length sequences is preferable. Furthermore, sequences may be missing from a transcriptome if they are not expressed at the time of sampling.

Our data indicate that the quality of the transcriptome, including the degree of fragmentation, and the resulting downstream peptide identifications can be improved to approximate that of a proteomic database through the careful selection of RNA-Seq read processing parameters before assembly. Databases made from transcriptomes that employed max_pct_stdev equal to 100 during normalization and/or read error correction identified a significantly greater number of peptides than databases constructed using max_pct_stdev equal to 10,000 and without error correction. A precise understanding of how the combined read processing methods improve transcriptome quality and the number of identified peptides is complex and beyond the scope of this paper. However, it is worth noting that each treatment affects the error content of reads. Sequencing errors occur on average in 0.1% of nucleotides in Illumina data [54], which can negatively impact assembly by increasing the size and complexity of de-bruijn graphs [55,56]. In addition, the presence of errors in a protein sequence database may lead to the omission of a peptide identification.

The primary means of error reduction in next-generation datasets are read quality trimming and error correction. Both methods generally improve transcriptome quality, although few studies focus on plants [25,43,47,57,58,59]. Quality trimming removes bases with a score below that of a selected threshold, commonly phred 20–35 [25,47,57,58,59]. This approach usually improves assembly quality [43,59], but in some cases may bias read content, and diminish assembly completeness [44,59]. Relaxed quality trimming at phred 5 may therefore be favored for some datasets and types of analyses [44,59]. In walnut seeds, selection of a trimming threshold of phred 35 or phred 5 yielded similar results, which is somewhat surprising given that studies comparing the effects of different thresholds on transcriptome quality usually reported one level as favorable over the other [44,59].

More in line with published data are the beneficial effects observed when Rcorrector was applied [39,55,59,60]. Rcorrector [39] utilizes de-bruijn graphs in conjunction with kmer counts to replace erroneous bases with correct ones. It operates on the assumption that erroneous kmers occur in lower frequencies than correct ones. Occasionally, this premise leads to the ill-correction of true kmers, particularly in low coverage regions. This introduction of new errors has also been shown to increase assembly breakpoints in some genome assemblies [56]. In walnut, all databases generated with the use of Rcorrector were high-performing, and transcriptome quality metrics were slightly better than those without error correction.

Less information is available regarding the effects of normalization parameters on assembly quality. Normalization serves primarily to remove redundant reads, but can also reduce errors [48,49]. One aspect of Trinity normalization [48] is the retention or elimination of reads with highly aberrant kmer coverage profiles via the maximum percent standard deviation parameter (-max_pct_stdev) [49,61]. When max_pct_stdev is set to 10,000 (default setting in current Trinity version), these reads are retained, while the reads are eliminated at lower settings of 100 or 200 (default settings in past Trinity versions). Reads with highly aberrant kmer coverage profiles are often erroneous, but, when correct, they provide critical support for the reconstruction of transcripts [49,61]. In walnut seeds, the removal of such reads was advantageous. However, care should be taken when selecting this value for additional datasets.

The significant increase in the total number of peptides identified when searching against databases constructed using Rcorrector or max_pct_stdev equal to 100 can be principally attributed to an increase in the identification of peptides which are also identified in the proteomic databases, due to an improvement in transcriptome assembly quality. In particular, highly abundant peptides, many of which align to seed storage proteins, were recovered in assemblies constructed using Rcorrector or max_pct_stdev equal to 100, but not in others (Table S6). Unique transcriptome-specific peptides were also identified in each of the 32 assemblies, regardless of the read processing methods used. Although a total of 132 such peptides were identified, the majority (106/132) are located at one of the termini of a fragmented or incorrectly assembled transcript, and are thus not fully tryptic. Most of the 26 fully tryptic peptides were identified due to the presence of at least one SAV, present either in the peptide of interest, or in the tryptic cleavage site adjacent to the peptide. The variation in SAVs across assemblies is a reflection of the combined effects of the complex trade-offs described above for each error reduction method.

The differential identification of transcriptome-specific peptides across assemblies is also due to inconsistencies in replicate assemblies of the same dataset. In some cases, assembly differences among replicates at the nucleotide level were exacerbated at the amino acid level due to differences in ORF finding. Trinity is non-deterministic; therefore, results from replicate assemblies are expected to be highly similar but not identical. Indeed, assembly statistics (Supplementary Table S4) indicate a high degree of consistency in quality across replicates. However, these aggregate statistics do not reflect the variability observed across specific homologous transcripts. The degree of variability across assemblies differed depending on the read processing conditions employed, with assemblies constructed using Rcorrector and max_pct_stdev set to 100 being the least variable. The increased reproducibility under these conditions further confirmed our selection of these methods for construction of the final, improved transcriptomic database, and future studies will focus on methods to further minimize variability. Alignment of raw RNA-seq reads to a reproducible reference transcriptome (or proteome, when available) will be useful to represent SAVs present in variable replicate assemblies as well as those present under different read processing methods.

## 5. Conclusions

For many tree nut species and other non-model organisms, protein sequences available in public repositories are insufficient for the construction of comprehensive MS/MS databases. This is particularly true for tree nut species without a sequenced genome. Using walnut as a model, we show that a de-novo assembled transcriptome can serve as a proxy protein database when a proteome is not available. In addition, the selection of appropriate RNA-seq read processing methods can improve transcriptome assembly quality and the number of peptides identified in downstream analyses. In both walnut and pecan, the use of read error correction and/or the reduction of reads with highly aberrant kmer coverage profiles led to the identification of a significantly greater number of peptides than when reads with aberrant kmer coverage profiles were retained and Rcorrector was omitted from the workflow. In addition, we provide insight into database construction when a genome and multiple proteomes are available. A proteomic database derived from the NCBI Eukaryotic Genome Annotation Pipeline yielded a greater number of peptides than those derived from the Maker and Braker pipelines. Searches against a 6FT database yielded only ten peptides not identified in searches against the NCBI proteome, suggesting that, for the analysis of tree nuts, novel peptide types, such as those from unannotated genes or alternative coding frames, are either not relevant or are lost in searching against a prohibitively large database. Given the impact of annotation on the number of identified peptides, additional studies which assess the utility of the manual annotation of a subset of target proteins will be important. Future studies will also seek to maximize the representation of variants in the transcriptome and proteome, as our work indicates that most peptides novel to the transcriptome originate from SAVs. Both RNA-Seq reads and genome resequencing data when available will be useful in this endeavor.

## Figures and Tables

**Table 1:** The number of residues, sequences, and identified peptides in each sized database. All database sizes include the contaminants database, 125 sequences, 40,028 residues.

	SIZED		UNSIZED		
Database Type	Number of Sequences	Number of Residues	Number of Sequences	Number of Residues	Number of Identified Peptides
NCBI Proteome	55,751	24,750,578	61,756	26,627,674	1275
Maker Proteome	32,621	13,113,315	76,087	26,627,669	1183
Braker Proteome	30,306	13,599,899	72,425	26,627,687	1156
Translated transcriptome	194,436	26,627,682	194,436	26,627,682	1200
Six-frame Translation Genome	172,954	679,657,178	NA	NA	719

## Supplementary Materials

The following are available online at https://www.mdpi.com/2079-7737/9/5/104/s1, Figure S1. Workflow of transcriptomic database construction, Figure S2. Plots of quality metrics for the 32 transcriptome assemblies. A. Total number of bases assembled B. Percent of reads aligned to the assembly C. N50 values D. Number of NCBI proteomes covered 90–100% by at least one transcript (100% sequence identity), Figure S3. Plot of the mean number of peptides identified across four transcriptomic databases constructed using different read processing conditions in pecan (*n* = 3). Bars represent standard error, Table S1. A list of all peptides identified in NCBI, Maker, and Braker databases. For each peptide, the number of spectral counts and the most parsimonious protein(s) inferred by each peptide are also reported, Table S2. A list of all peptides identified in databases built from the NCBI proteome and the transcriptome assembled using published methods 25. For each peptide, the number of spectral counts and the most parsimonious protein(s) inferred by each peptide are also reported, Table S3. ANOVA results using Tukey’s adjustment for multiple comparisons. Table S4. Quality metrics for 32 walnut transcriptome assemblies, TableS5. The presence of sequences of all uniquely identified peptides from the three-way comparison of the NCBI, Maker, and Braker databases in all three proteomes, Table S6. A list of all high-abundance peptides (at least 100 spectral counts) identified in at least one transcriptome assembly. Spectral counts listed for each of the four transcriptome assembly replicates for each set of read processing conditions. Data S1. A fasta file of the translated improved transcriptome.
