# Supplementary figures and images for: The Utility of Genomic and Transcriptomic Data in the Construction of Proxy Protein Sequence Databases for Unsequenced Tree Nuts
Source: Biology (Basel). 2020 May 19;9(5):104. doi: 10.3390/biology9050104 (PMC7284556; doi:10.3390/biology9050104)

**A**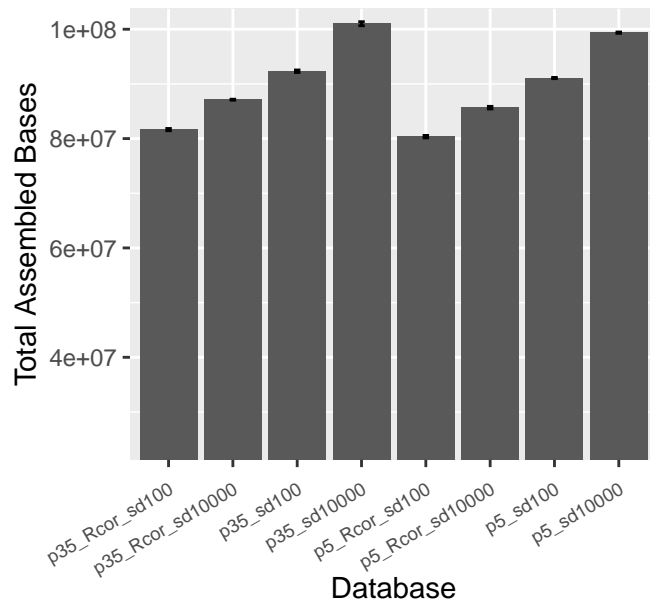**B**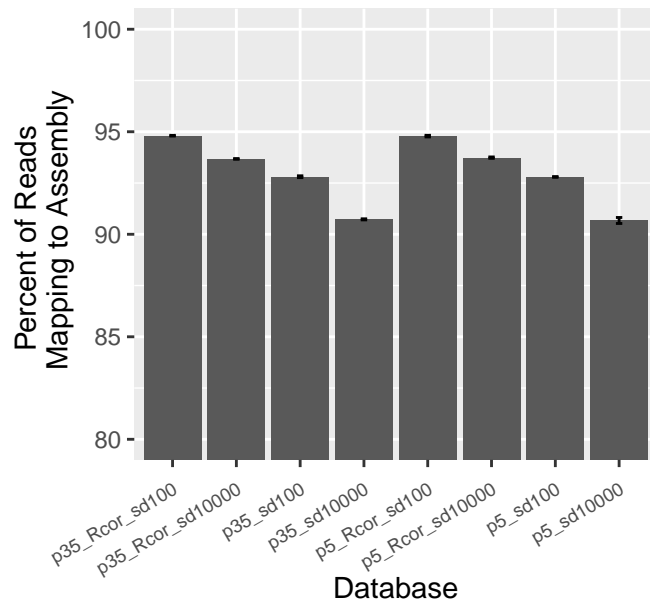**C**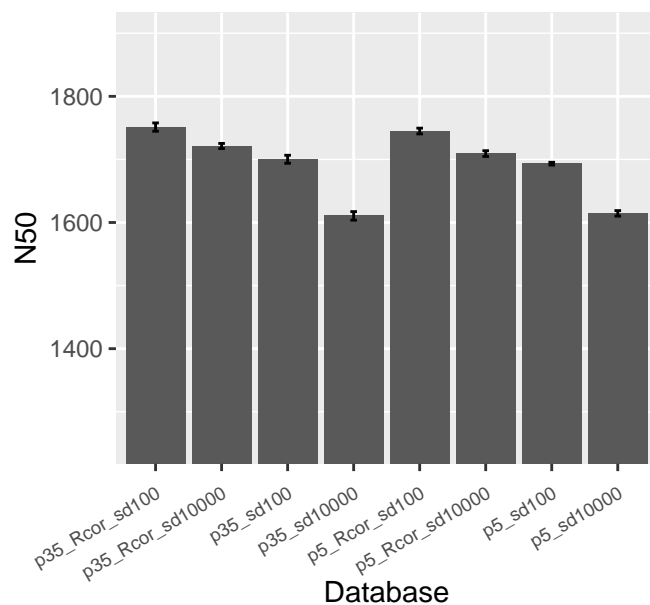**D**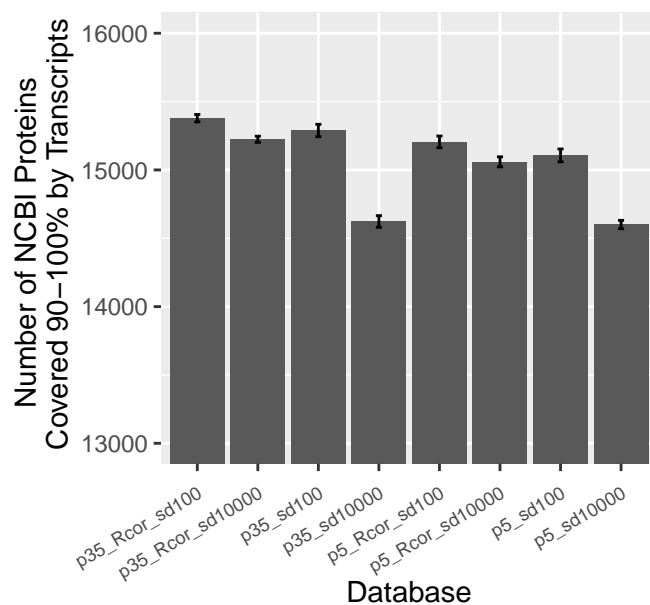

Supplement: Supplementary file 1 [file biology-09-00104-s001.zip › Supplementary_Files_Pirone-Davies_final/FigureS2.pdf]

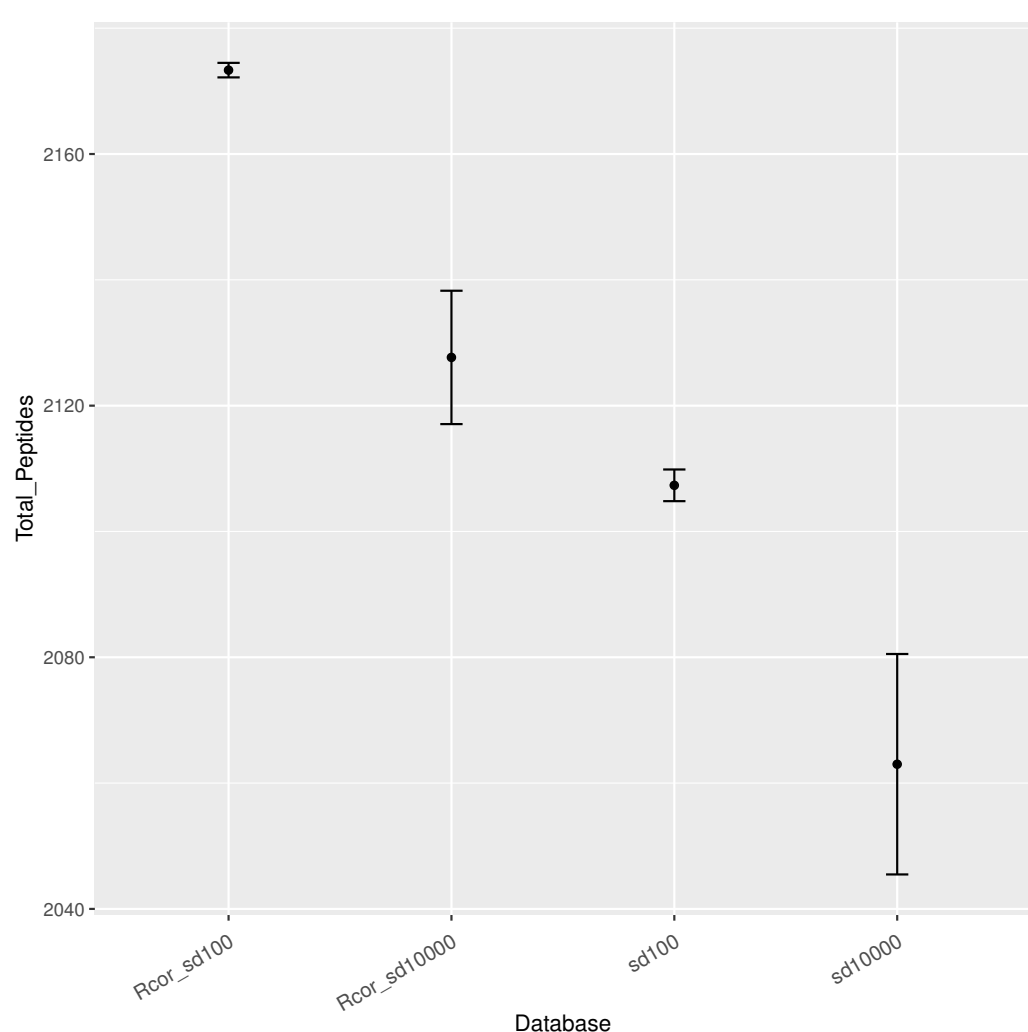

Supplement: Supplementary file 1 [file biology-09-00104-s001.zip › Supplementary_Files_Pirone-Davies_final/FigureS3.pdf]

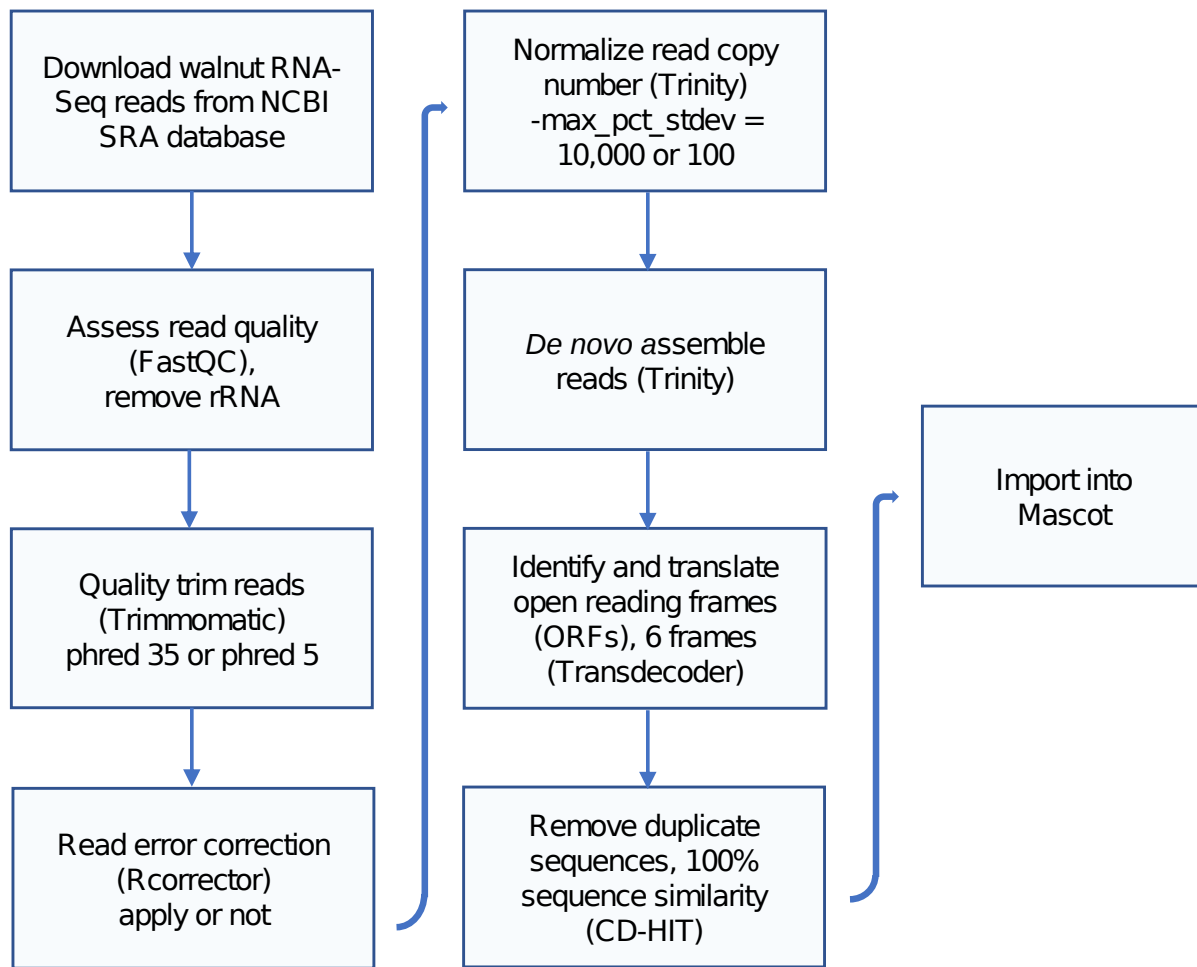

Supplement: Supplementary file 1 [file biology-09-00104-s001.zip › Supplementary_Files_Pirone-Davies_final/FigureS1.pdf]
